# Diversity of Phosphorus‐Solubilizing Microbes Isolated From Different Cropping Systems of Zimbabwe for Use as Biofertilizers With Rock Phosphate

**DOI:** 10.1002/mbo3.70065

**Published:** 2025-10-13

**Authors:** Grace Kanonge, Mazvita S. Chiduwa, Pardon Muchaonyerwa

**Affiliations:** ^1^ Department of Research and Specialist Services (DRSS) Soil Productivity Research Laboratory (SPRL), Chemistry and Soil Research Institute (CSRI), MLAWFRD Marondera Zimbabwe; ^2^ College of Agriculture, Engineering and Science (CAES), School of Agricultural, Earth, and Environmental Sciences (SAEES) University of KwaZulu Natal Pietermaritzburg South Africa; ^3^ International Maize and Wheat Improvement Center (CIMMYT), c/o ICRISAT, Sustainable Agrifood Systems Program (SAS) Lilongwe Malawi

**Keywords:** bacteria, biochemical properties, bioinoculant, phylogeny, solubilization index

## Abstract

Soil phosphorus deficiency and the high cost of mineral fertilizers necessitate research into alternative strategies. Inoculating seeds with adapted phosphorus‐solubilizing microorganisms (PSMs) could be a cost‐effective option. This study explored diversity, including phenotypic and genotypic characteristics of PSMs from selected soils and cropping systems of Zimbabwe, for coapplication with rock phosphate (RP). Culturable PSMs were isolated from preincubated or cowpea rhizosphere soil. Over 91% of the 37 isolates (PSM1–PSM37) were bacteria, while 8% were fungi. Diversity was higher in Dorowa (*H′* 2.99; DMn 8.49) than that in Marondera (*H′* 2.85; DMn 7.57), and under groundnut and maize (*H′* 3.26) than other crops. Some PSMs occurred only in Marondera (8%) and Dorowa (14%). The P solubilization index on RP‐amended Pikovskaya medium, ranged between 1.00 and 15.9. Sixty‐five percent of the best 28 isolates were Gram‐negative cocci or bacilli, while 35% were Gram‐positive cocci. A dendrogram based on morphological, biochemical, and functional characterization grouped the isolates into two major clusters and four subgroups. On the basis of 16S recombinant DNA analyses, the *Bacillus* genus predominated (61%), with the highest P solubilization capacity (*Bacillus amyloliquefaciens*), while the rest (39%) were *Enterobacter*, *Microbacterium*, *Paenibacillus, Klebsiella*, *Priestia*, *Acinetobacter*, *Nocardioides*, and *Kocuria* genera. In conclusion, studied soils harbor diverse PSMs that solubilize RP, indicating their potential to develop affordable bioinoculants for improved productivity on P‐limited soils. The study pioneers the discovery of diverse PSMs native to Zimbabwean soils. Further studies are required to evaluate PSM–RP efficacy with various crops under glasshouse and field conditions and benchmark with conventional fertilizers.

## Graphical abstract

Thirty‐seven potential culturable P‐solubilizing bacteria candidates were isolated from Zimbabwean soils and can unlock P in rock phosphates, offering an alternative affordable source of P for resource‐constrained farming communities.

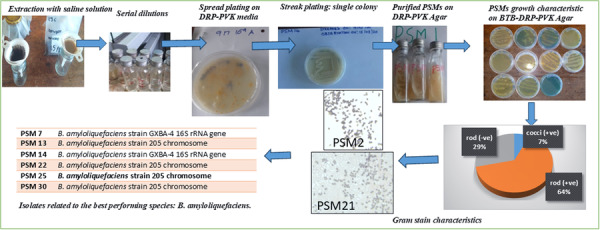

## 1 Introduction

Crop productivity is limited by inherent low nitrogen (N) and phosphorus (P) levels in the majority of the agricultural soils of sub‐Saharan Africa, including Zimbabwe (Mapfumo and Giller 2001; Dimkpa et al. 2023). Continuous cereal monocropping and nutrient mining have created the need for the application of large amounts of external nutrient inputs to realize sustainable yields and maintain soil quality. The potential inputs include inorganic fertilizers, organic nutrient resources (e.g., animal manures and composts), green‐manure legume crops, intercrops, rotational legume crops, anthill soil, and nutrient‐rich rock deposits (Dhliwayo 1998; Mtangadura et al. 2017; Tumbure and Schmalenberger 2019). Despite this wide range of options, there are some limitations in their use, which result in low crop yields, far below 1.0 Mg ha^−^^1^ for legumes and a continuing decline in soil health (Mafongoya et al. 2006; Kanonge et al. 2015). Nitrogen and P supply technologies are essential for increased crop growth and productivity to transform the food systems by 2030, fighting malnutrition and contributing to the eradication of extreme poverty (Maley and Peachey 2017; Oluwole et al. 2023). Balanced and affordable crop fertilization strategies can help in breaking the poverty cycle, especially for resource‐constrained households.

There has been considerable effort to enhance the production of nutrient‐dense legumes through the application of biological nitrogen fixation (BNF) and the rhizobia inoculant technologies in Zimbabwe and other countries (Catroux et al. 2001; Herridge et al. 2008; Lindström et al. 2010). For effective BNF, with or without inoculation, P is often a limiting factor. Provision of P is crucial in legume cropping systems, since P is a component of nucleic acids, enzymes, coenzymes, nucleotides, and phospholipids (Vassilev et al. 2006; Kolodiazhnyi 2021). Phosphorus accounts for between 0.2% and 0.8% of the dry weight of plants, varying with nutrient supply patterns in the cropping system (Sharma et al. 2007). Shortage of P decreases the nutrient uptake efficiency of other inputs, including N, and causes poor nodule formation and nitrogen fixation in legumes (Somasegaran and Hoben 1994; Tittonell et al. 2007). Phosphorus fixation, especially under low pH conditions, such as those found in Marondera district of Zimbabwe, reduces the availability of P and makes annual applications of P inputs a prerequisite for sustainable crop productivity (Barrow and Hartemink 2023; Audette et al. 2016; Weeks and Hettiarachchi 2019). Solutions are required to improve P availability, to maximize the benefits from the existing N‐fixation microbial biostimulants and to improve the N‐fixation capacity of legumes grown in Zimbabwe. While rock phosphate (RP) may be locally available, its poor solubility in soil limits its direct application.

The world has approximately 71 billion metric tons (bmt) of RP reserves (US 2021). The phosphate in these rocks occurs in the form of carbonate apatite [3Ca_3_(PO_4_)_2_·CaCO_3_], hydroxyapatite [Ca_10_(PO_4_)_6_(OH)_2_], fluorapatite [Ca_10_(PO_4_)_6_F_2_], and sulpho‐apatite [3Ca_3_(PO_4_)_2_·CaSO_4_] (Audette et al. 2016). Although the igneous Dorowa Rock Phosphate (DRP) used in the manufacture of P fertilizers is abundant in the Buhera district of Zimbabwe, it is highly insoluble. Biological solubilization, involving the coupling of phosphorus‐solubilizing microbes (PSMs) with the insoluble RPs, with or without organic manure application, has proved to be a promising technology in other countries (Whitelaw et al. 1999; Sharma et al. 2007; Saif et al. 2014; Ughamba et al. 2025). The effectiveness of phosphorus‐solubilizing bacteria (PSB) with or without RP has been confirmed (Akbari et al. 2010; Anzuay et al. 2015; Bahadir et al. 2018; Cheng et al. 2023). Janati et al. (2023) reported that the community structure of PSMs, their production of auxins and siderophores, which contribute to their P‐solubilizing competence, are mostly influenced by the climate of the area. There is no documented work on PSMs isolated from Zimbabwean soils. The isolation of native PSMs that are adaptable to local Zimbabwean conditions could significantly contribute to the development of P biofertilizers, especially when used with DRP. The use of microbe‐based P‐solubilizing technology could significantly complement efforts of the only current in‐country producer of N‐fixing legume rhizobia inoculants, namely, Legume Inoculant Factory, which is based in Marondera, Zimbabwe (Anzuay et al. 2015; Lindström et al. 2010; Bahadir et al. 2018). The existing infrastructure can be used for the production of P‐solubilizing microbial (PSM) inoculants, which may contribute to the reduction of the country's fertilizer importation costs. The PSM biofertilizer could therefore be a promising technology, which is relatively cheaper, especially when applied with the local RP. Globally, green technology is being recommended (Baumgart‐Getz et al. 2012; Donkor et al. 2019; H. Mao et al. 2021) and PSM biofertilizers are among the technologies which could reduce environmental hazards (Galal et al. 2001). PSMs also play a significant role in the biogeochemical cycling of soluble and insoluble P in agricultural ecosystems by converting insoluble P into soluble P, which then becomes accessible to plants (Acevedo et al. 2014; Li et al. 2017), promoting soil biodiversity. The success of this approach will depend on the successful isolation of effective microorganisms.

A wide range of PSMs have been isolated from various ecosystems, including water bodies and agricultural soils. For example, PSMs of diverse genera were isolated from chickpea rhizosphere soil in Ethiopia and found to be effective in P solubilization and N_2_ fixation (Mulissa et al. 2016). Fungi from the *Penicillium* and *Aspergillus* genera, along with bacteria belonging to the *Bacillus* and *Pseudomonas* genera, are among the most frequently reported PSMs in the literature (Mohamed et al. 2014). Other less common phosphorus‐solubilizing bacteria include genera, such as *Rhodococcus*, *Arthrobacter*, *Serratia*, *Chryseobacterium*, and *Phyllobacterium* (Wani et al. 2005), as well as *Azotobacter* (Kumar et al. 2001), *Xanthomonas* (De Freitas et al. 1997), and the *Enterobacter*, *Pantoea*, and *Klebsiella* genera (Chung et al. 2005). These microbes use different mechanisms, including pH reduction and the release of organic acids, which would solubilize the RP, chelate the ions that could precipitate with phosphates (e.g., Al and Fe) and compete with the phosphates for sorption sites (Ingle and Padole 2017; Ahash et al. 2025). The success of any inoculant product depends on a range of factors, including soil type, host–species compatibility, microbial population, or abundance, the presence of competing indigenous bacteria, and the genetic stability of the microbe under consideration (López‐García et al. 2002; Farooq and Vessey 2009). Considering that the community structure of PSMs and their efficiency are dependent on climatic and soil factors, there is a need to isolate and characterize locally adaptable P‐solubilizing bacteria. The findings of such a study would guide the development of a P biofertilizer technology for Zimbabwean conditions. The objectives of the current study were to (i) determine the occurrence and diversity of PSMs in selected soils with different land use or cropping history from two contrasting districts of Zimbabwe and to (ii) screen PSM isolates for the capacity to solubilize phosphorus in DRP, as a first step toward identifying locally adaptable candidate strains for producing phosphate‐based inoculants for use with the RP.

## 2 Materials and Methods

### 2.1 Site Description

Virgin and cultivated soils from 11 fields in Marondera district (near the Legume Inoculant Factory) and 10 fields in Buhera (close to DRP mine) were used in this study. The Legume Inoculant Factory, established in 1962, is sited at the Soil Productivity Research Laboratory (SPRL), domiciled at Grasslands Research Station, in Marondera (18°10.971′ S; 031°29.871′ E) (Raimi et al. 2021). The laboratory (SPRL) was used for all laboratory experiments and microbial culture procedures under the SPRL‐Microbial Culture Bank. The DRP mine is located about 90 km west of Mutare (19°3′49″ S; 31°45′45″ E). The selected fields had no history of bioinoculant application. Considering the importance of climate on PSM community structure and P solubilization efficiency, sites in contrasting agroecological zones were required. Marondera district falls in Natural Region (NR) IIB, characterized by an annual rainfall average ranging between 700 and 1000 mm. In contrast, Buhera district is in NR III, with an annual rainfall average between 550 and 700 mm. The area is characterized by midseason dry spells and high temperatures (Nyamapfene 1992; Amin et al. 2015). The length of the growing period for maize varies between the two regions with 105–120 and 120–135 days in Marondera and Buhera, respectively. However, the two areas have patches of similar soils dotted across the districts, including the vertisols, siallitic, fersiallitic, paraferralitic, and orthoferralitic groups (Nyamapfene 1992; Food and Agriculture Organization (FAO) of the United Nations 2004). Vegetation abundance, total rainfall, and crop production potential decline from NR I to NR V across Zimbabwe (Mugandani et al. 2012). Agricultural practices in Marondera and Buhera are characterized by mixed crop and livestock production. In Marondera, major crops are tobacco and maize. Other crops include soybean, groundnut, wheat, and common bean. Major crops in Buhera include drought‐tolerant and traditional grains, such as cowpea, sorghum, and finger millet. Livestock production is dominated by cattle rearing in both Marondera and Buhera, with the major difference being the practice of pasture grazing and pen fattening in the former, while in the latter, communal rangeland grazing and extensive beef ranching predominate (Roth and Haase 1998; Musendo et al. 2022). Most fields in both Marondera and Buhera have sandy to sandy loam soils, with scattered patches of black or red clays (Chikodzi et al. 2013). They therefore represent most soils cropped under smallholder agriculture in Zimbabwe. We did not explore all types of soils found in Zimbabwe, since this is one of the first studies on isolation and evaluation of PSMs from Zimbabwean soils. Therefore, the proof of occurrence and detailed characterization were prioritized over extensive isolation.

### 2.2 Soil Sampling and Preparation

Soil samples were collected from 10 farmers' fields in each of the districts, in addition to an on‐station research plot at SPRL. Composite soil samples of 1 kg and bulk 9 kg weights (0–20 cm depth) were each aggregated from 10 sampling positions, using the zig‐zag sampling method (Kumar et al. 2001) in each ≤ 1.0 ha plot with an auger and a spade, respectively. The samples were air dried and sieved (< 2 mm).

### 2.3 Soil Characterization

The soils were analyzed for pH (calcium chloride), soil texture (hydrometer method), available P (Olsen method); organic carbon (modified Walkley Black method); total exchangeable bases (acidified ammonium acetate method); total N and P (acid digestion with hydrogen peroxide and colorimetry method); and mineral N (potassium sulfate extraction followed by colorimetry) (Okalebo et al. 2002; Anderson and Ingram 1993).

### 2.4 Isolating PSMs

The PSMs were isolated using both the soil‐direct and the bait plant methods before further characterization (Cheng et al. 2023).

#### 2.4.1 The “Soil‐Direct” Method

A 10‐g soil sample was drawn from each composite sample and placed into a 1000‐mL polythene bottle, followed by the addition of 990 mL of sterile saline solution (0.85% w/v NaCl), which had been sterilized by autoclaving at 121°C and 1 atm. The soil was moistened to introduce a uniform moisture environment and preincubated at 28°C for 7 days to ensure the activation of dormant microbial organisms, since samples were collected under dry conditions. The suspension was shaken for 30 min at 120 rpm using a multiorbital functional shaker‐PSU‐20i, followed by filtration and centrifugation to generate a clear solution. The PSMs in each sample were determined using the plate count method, with three replicates, on selective P medium {Pikovskaya (PVK)} Petri dishes after serial dilutions from 10^−^^3^ to 10^−^^9^. The PVK medium contains 10 g glucose, 5 g Ca_3_(PO_4_)_2_, 0.5 g (NH_4_)_2_SO_4_, 0.2 g NaCl, 0.1 g MgSO_4_·7H_2_O, 0.2 g KCl, 23 g yeast extract agar, 0.0001 g MnSO_4_·H_2_O, and 0.0001 g FeSO_4_·7H_2_O per liter of sterile water (Nautiyal 1999).

The plates (Petri dishes) were incubated at 28°C ± 2°C for 5 days to trigger multiplication of microbial cells. This was followed by visual and microscopic observations to record the morphological characteristics of the microbes. Identified fungi were morphologically characterized but immediately destroyed using 95% ethanol. Further work proceeded with bacteria, since the SPRL is currently only meant for bacterial work and has operational standards that do not allow cultivation, multiplication, or storage of fungal matter, to minimize fungal contaminations. A Stuart colony counter, model SC 6, was used to aid the enumeration of colonies in the plates at day 5. Bacteria colony‐forming units (CFU g soil^−^^1^) were enumerated based on the most diluted and representative level for each sample, since their cultural characteristics were not known yet. The PSM population was thus calculated using Equation (1):

(1) $$\mathrm{CFU}\;g\;{\mathrm{soil}}^{‐1}=\frac{\mathrm{number}\;\mathrm{of}\;\mathrm{colonies}\times \mathrm{dilution}\;\mathrm{factor}\;(3\;\mathrm{replicates})}{\mathrm{volume}\;\mathrm{of}\;\mathrm{aliquot}}.$$

Isolates that produced clear halo zones or grew in abundance on PVK agar were selected and designated names starting from PSM1. The halo zone diameter was measured for each identified PSM. A loopful of each pure bacterial colony (from single colonies cultivated on fresh PVK agar plates) was transferred to agar slant bottles in three replicates for further storage at 4°C under asceptic sterile conditions in a laminar flow cabinet. Subculturing phosphate‐solubilizing bacteria (PSB) was done every 3 months to maintain pure viable stocks in the SPRL‐Microbial Culture Bank.

#### 2.4.2 The “Bait Plant” Method

Surface‐sterilized cowpea seeds (variety‐CBC 2) were pregerminated at 28°C ± 2°C for 3 days (Alam et al. 2002) before planting three seedlings in 3 kg soil in 3‐L plastic pots. This was replicated three times per soil sample. Cowpea was selected as the bait plant since the study was focused on identifying PSMs that improve cowpea productivity. Having cowpea roots in the rhizosphere attracts and promotes the growth of those bacteria that would form a relationship with cowpea. Pots were watered and maintained at field capacity moisture content with distilled water throughout the growing period in a greenhouse. At 45 days, after planting plants were carefully uprooted from the soil at field capacity moisture and sampled together with all soil that had adhered to the roots. The PSMs were isolated from the rhizosphere soil as detailed for the “soil‐direct” method. The PSMs identified were designated numbers continuing from the list generated from the “soil‐direct” method. Isolates similar to those identified in the “soil‐direct” method were classified accordingly as already established. These were identified through their morphological characteristics. All work on further characterization was done with bacteria only, despite their designation as PSMs, since the initial isolation included some fungal isolates.

Relative frequencies (% of total) of bacterial PSMs isolated from each area and under different land use practices were computed.

### 2.5 Characterization of the Isolates

#### 2.5.1 Halo Zone Production and Growth of PSB on DRP‐Amended PVK Agar

A preliminary experiment was conducted to compare the growth of the PSMs on original PVK containing calcium phosphate (CAP–PVK) and on DRP‐amended PVK media (DRP–PVK). The DRP–PVK media were prepared by combining all reagents for PVK agar as described for the “soil‐direct” method, but the CAP was replaced with raw DRP. The raw RP used in this study was collected from Dorowa Mine. It was ground and passed through a 1.0‐mm sieve and analyzed for total P using digestion with sulfuric acid and hydrogen peroxide, followed by colorimetric measurements (Okalebo et al. 2002). The DRP was found to contain 2.7% total P. Three different isolates (PSM1, PSM7, and PSM27), representing the different classes of bacteria isolated, were selected based on colony morphology (color, shape, and diameter) and halo zone diameter. The results showed that the growth of the PSMs varied among the isolates but did not depend on the P source.

An experiment was done with only DRP–PVK medium to determine the growth of all the bacterial PSMs that maintained the identified morphological characteristics and could be cultured and purified after storage at 4°C for 4 weeks. Twenty‐eight isolates were found to be consistent and hence these were considered for this investigation and subsequent investigations. The RHIZO 276 (*Rhizobium leguminosarum*), a known N‐fixing rhizobia strain was taken from the SPRL‐Microbial Culture Bank and provided similar media conditions as the unknown PSM isolates. The respective isolates were spot inoculated onto DRP–PVK media, in two replicates and incubated at 28°C ± 2°C for 7 days. Single colonies were studied for morphological characteristics, including color and elevation. The presence or absence of a halo zone around the colony was recorded after 5 days of incubation and on day 6 using a colony counter/viewer2. The Phosphate Solubilization Index (PSI) was computed using Equation (2) (Farooq and Vessey 2009).

(2) $$\mathrm{PSI}=\frac{\mathrm{colony}\;\mathrm{diameter}+\mathrm{halo}\;\mathrm{zone}\;\mathrm{diameter})}{\mathrm{colony}\;\mathrm{diameter}}.$$

A second experiment was set up to quantitatively determine the number of days taken to solubilize P in DRP–PVK agar plates. Bromothymol blue (5 mL of 0.5% BTB/L) was included in the medium preparation (BTB–DRP–PVK agar) before pouring into respective Petri dishes for the 28 PSM isolates in three replicates. Pure cultures of the PSM bacteria were streaked onto BTB–DRP–PVK agar plates following sterile and aseptic procedures and incubated at 28°C ± 2°C for a period of 10 days. The RHIZO 276 (*R. leguminosarum*) (a fast grower) and RHIZO 267 (*Bradyrhizobium diazoefficiens*) (a slow grower), taken from the SPRL‐Microbial Culture Bank, and the blank plates were included as controls. The plates were examined daily to track the color change from blue to yellow. Bromothymol blue is a pH‐sensitive indicator and was therefore expected to change because of organic acid production as a mechanism of RP solubilization. Plates were discarded once the yellow color was observed. The average number of days to color change was computed.

#### 2.5.2 Biochemical Characterization of the PSM Isolates

Biochemical characterization was done for the 28 PSM isolates and the two known rhizobia isolates (RHIZO276 and RHIZO267). The isolates were characterized by using the Gram‐stain method (Somasegaran and Hoben 1985). The isolates' capability to hydrolyze sugars was assessed using phenol red carbohydrate (glucose/maltose/sucrose or fructose) broth and a Durham tube for gas detection (Vos et al. 2011). The catalase test was conducted using 30% H_2_O_2_ (Anand et al. 2016). The citrate test was done using *Simmons Citrate agar* (Vos et al. 2011). The indole test was used to screen the PSM bacteria for the ability to degrade the amino acid tryptophan using Kovac's reagent as outlined in Bergey's manual for systematic bacteriology (Vos et al. 2011).

### 2.6 Phylogenetic Identity and Analyses of PSM Isolates Using 16S recombinant DNA (rDNA)

The 28 PSM isolates and the two known rhizobia standard strains from the SPRL‐Microbial Culture Bank were genotypically characterized by sequencing their 16S rDNA using universal primers (sequence 5′–3′): 27F (AGAGTTTGATCMTGGCTCAG) and 1492R (CGGTTACCTTGTTACGACTT) (Lane et al. 1988) at Inqaba Biotechnical Industries, South Africa. Genomic DNA was extracted from the respective cultures using the Quick‐DNA Fungal/Bacterial Miniprep Kit, Zymo Research Catalogues No. D6005 (Zymo Research 2016). The 16S target region was amplified using OnTaq Quick‐Load 2x Master Mix (NEB Catalogue No. M0486) with the primers described above (Zhang et al. 2020; D. P. Mao et al. 2012). The PCR products were run on a gel, which was then extracted with Zymoclean Gel DNA Recovery Kit, Zymo Research Catalogue No. 4001 (Zymo Research 2016). The extracted fragments were analyzed on the ABI 3500xl Genetic Analyzer (Sasitaran et al. 2017; Applied Biosystems 2011). The CLC Bio Main Workbench v7.6 was used to analyze the *ab1* files generated by the ABI 3500XL Genetic Analyzer (Liu and Peter Di 2020; Smith 2015). The obtained 16S rRNA gene sequences were edited using the ChromasPro software (version 2.6.6). The pairwise nucleotide percent identity of the new sequences was calculated using MEGA 11.0.13 software (Tamura et al. 2021). Results were obtained by a BLAST search in the National Center for Biotechnology Information (NCBI) database (Altschul 1997), at 99%–100% similarity. The identified sequences were uploaded to the GenBank nucleotide sequence database and allocated accession numbers.

## 3 Statistical Analyses

Data on the characterization of soils or PSMs were presented after calculation of averages based on replicates for each parameter, followed by computation of the SE of means. All quantitative data generated from structured experiments, including PSI, were subjected to one‐way Analysis of Variance using Genstat 20th Version. Means were separated using Fischer's Protected Least Significant Difference (Williams and Abdi 2010).

PSM isolate occurrence data was transformed {√(*x* + 0.5)} followed by analysis with the alpha diversity, Shannon Wiener (*H′*) index, using the Paleontological Statistics (PAST) package version 4.02 (Hammer et al. 2001), to understand the species diversity under different land use/cropping histories. PAST is a free statistical software package used for data analysis, with various functions for data manipulation, plotting, univariate and multivariate statistics, ecological analysis, and spatial analysis (Hammer et al. 2009). Species richness (number of species present) and abundance (number of individuals per species) were described by the Menhinick index (DMn), while diversity was rated by the Shannon index, which has a maximum of 5, with an increasing value as species diversity increases. Species richness is computed by dividing the number of species observed by the total area of the defined ecosystem. Simpson's index (*D*), representing the probability that two randomly chosen individuals belong to different species (Morris et al. 2014), and the Menhinick Index, which estimates species richness as the number of species in a sample divided by the square root of the number of individuals in the sample (Mulya et al. 2021), were used to understand the diversity of PSMs from different fields samples. The equations below show the formulas for the calculation of the indices used.

(3) $$\mathrm{Shanon}\;\mathrm{Index}\;\left(H\prime \right)=\sum_{i=1}^{s}p_{i}\;\ln\;p_{i},$$

(4) $$\mathrm{Menhinick}\;(\mathrm{DMn})=\frac{\mathrm{Si}}{\surd N},$$

(5) $$\mathrm{Simpson}\;(D)=1-\left(\frac{\Sigma n(n-1)}{N(N-1)}\right).$$

In the above equations, *p* is the proportion (*n*/*N*) of individuals of each species found, ln is the natural log, Σ is the sum of the calculations, and *s* is the number of species (Shannon and Weaver 1949; Menhinick 1964; Kunakh et al. 2023; Simpson 1949). Despite there being several diversity indices, the Menhinick index was selected because it is less influenced by sample size compared with the Margalef index, while the Shannon index, representing the uncertainty about “the identity of an unknown individual,” is independent of sample size and can better differentiate the diversity between systems (Morris et al. 2014; Kunakh et al. 2023). Simpson's index (*D*) was considered to further comprehend the findings because it is easy to understand, although it is heavily weighted by dominance (Simpson 1949; Hill et al. 2003). The standard error (SE), at 95% confidence interval, of the indices was computed using the indices' data generated from the PAST Software using the formula shown below (Moore et al. 2009); Chandler et al. 2019).

(6) $$\mathrm{Standard}\;\mathrm{error}\;(\mathrm{SE})=\frac{\left(\mathrm{upper}\;\mathrm{limit}-\mathrm{lower}\;\mathrm{limit}\right)}{3.92}.$$

The indices were then compared between the crop/land use treatments by percentage increment or decrease. The data set was not balanced as it was generated from a survey. The occurrence of the PSMs was not predetermined. Diversity indices lack a well‐defined probability distribution, and as such, no further statistical analyses were done on the indices obtained (Mander et al. 2012; Gatti et al. 2020). Multivariate cluster hierarchical analyses were conducted based on selected morphological properties, all biochemical characteristics, halo zone diameter, and the P solubilization index (Saraçli et al. 2013), using SPSS 29th version.

## 4 Results

### 4.1 Chemical Properties and Land Use History of Soils Sampled for PSM Isolation

In both districts, some fields were previously cropped to maize or a legume crop (cowpea, bean, or groundnut). Dorowa soils had higher soil P and lower SOC than those of Marondera, with no differences in terms of soil pH and mineral N between the areas (Table 1). Eighty percent of the soils sampled were medium‐grained sandy soils and generally acidic, with pH values ranging between 4.0 and 5.7. There were differences in pH associated with previous field history: fallow, vlei, cowpea (*Vigna unguiculata* L.), sunflower (*Helianthus annus* L.), and research field soils had lower pH than that under sugar bean (*Phaseolus vulgaris* L.). The research field had the lowest pH followed by that previously planted to sunflower and the previously fallow soil. Soil available phosphorus (P) levels were highly variable ranging between 26 and 264 ppm, with undisturbed (virgin) fields having 26–59 ppm. Soils under sugar bean had the highest available P levels followed by groundnut (*Arachis hypogaea* L.), cotton (*Gossypium herbaceum* L.), and sorghum (*Sorghum bicolor* L.), with the vlei soil having the lowest. Mineral N in soils studied averaged 17.4 and 19.0 mg kg^−^^1^ soil in Dorowa and Marondera, respectively (Table 1). Soil organic carbon ranged between 0.31% and 5.4% (virgin land), while exchangeable bases (meq/100 g) were between Ca 2.22–5.58, Mg 0.22–0.69, and K 0.84–2.84 (Table 1).

**Table 1:** Selected soil properties as affected by area and previous crop (mean ± SEM).

Unit		mg kg^−^^1^		%	meq/100 g		
Factor	Soil pH	Available P	Mineral N	SOC	Exch Ca	Exch Mg	Exch K
*Area*							
Dorowa	4.91 ± 0.11	139 ± 23.2	17.4 ± 4.38	0.63 ± 0.12	4.20 ± 1.02	0.42 ± 0.09	1.97 ± 0.53
Marondera	4.96 ± 0.13	84.5 ± 16.6	19.0 ± 3.85	1.52 ± 0.38	2.96 ± 0.28	0.52 ± 0.09	1.21 ± 0.16
*Previous land use*							
Cotton	5.00	176	42.0	0.34	2.22	0.22	0.84
Cowpea	4.90	63.0	38.0	3.10	2.75	0.65	1.24
Fallow	4.60 ± 0.06	72.0 ± 21.5	8.33 ± 4.37	0.450 ± 0.08	5.58 ± 2.89	0.28 ± 0.05	2.40 ± 1.21
Groundnut	4.85 ± 0.25	179 ± 17.0	14.5 ± 0.50	0.575 ± 0.01	2.59 ± 0.48	0.23 ± 0.04	0.92 ± 0.11
Maize	4.95 ± 0.19	95.2 ± 18.1	16.3 ± 5.40	0.833 ± 0.23	3.28 ± 0.68	0.60 ± 0.16	1.44 ± 0.39
Sorghum	5.10	165	15.0	0.550	3.03	0.67	1.13
Sugar bean	5.35 ± 0.35	230 ± 34.0	29.0 ± 11.0	1.12 ± 0.14	4.68 ± 1.17	0.69 ± 0.16	2.84 ± 1.20
Sunflower	4.30	158	21.0	2.15	2.73	0.47	0.96
Virgin	5.20 ± 0.00	41.0 ± 9.64	16.0 ± 7.21	0.99	2.80 ± 0.17	0.40 ± 0.04	1.00 ± 0.04
Vlei	4.90	32.0	9.00	5.40	4.54	0.38	2.61

*Note:* Soil pH (CaCl_2_).

Abbreviations: Exch., exchangeable; Min‐N, incubated mineral N; SOC, soil organic carbon.

The soils previously under cotton had the highest mineral N followed by those under cowpea and sugar bean, with fallow and vlei having the lowest (Table 1). The SOC was highest in soils previously under cowpea and sunflower, followed by sugar bean, virgin land, and maize (*Zea mays* L.), with the lowest being under cotton.

### 4.2 Occurrence and Abundance (CFU g Soil^−^^1^) of PSMs in Soil

PSM8 occurred in all soils, followed by PSM1 with 86% occurrence, and PSM3 in 81% of the soils studied (Table 2). Eleven isolates were each recovered from only one soil sample, through the bait plant method. All isolates that were recovered directly from the soil occurred in at least 10 soil samples, except PSM6, PSM7, PSM14, and PSM15, which were recovered in between 3 and 5 soil samples (Table 2). Isolates obtained through the bait plant method had low repeated occurrence across soil types. Only five isolates (PSM16, PSM20, PSM22, PSM26, and PSM27) were recovered from four soil samples (Table 2).

**Table 2:** Occurrence of PSMs in different soils.

Isolate	Number of soils in which PSMs occurred	% Occurrence
PSM8	21	100
PSM1	18	86
PSM3, PSM12	17	81
PSM2	16	77
PSM10, PSM13	15	72
PSM9	13	62
PSM5	11	52
PSM4, PSM11	10	48
PSM14	5	24
PSM7, PSM16, PSM20, PSM22, PSM26, PSM27	4	18
PSM6, PSM15, PSM25, PSM33	3	14
PSM17, PSM18, PSM21, PSM34	2	9
PSM19, PSM23, PSM24, PSM28, PSM29, PSM30, PSM31, PSM32, PSM35, PSM36, PSM37	1	5

Abbreviation: PSM, phosphorus‐solubilizing microorganism.

The highest number of colonies was recorded for PSM6, PSM7, PSM14, and PSM15, with beyond 1.50 × 10^9^ CFU g^−^^1^ soil. These four PSMs produced copious minute colonies. Among isolates with bigger colonies, the population ranged from 7.26 × 10^7^ (PSM10) to 1.32 × 10^9^ CFU g^−^^1^ (PSM4) (Table 3).

**Table 3:** Population of PSMs (CFU g soil^−^^1^) in source soil.

Strain	*N* a	CFU g soil^−^^1^ (× 10^7^)	SEM × 10^7^
PSM1	13	94.60	19.42
PSM2	16	469.10	79.07
PSM3	17	283.12	130.72
PSM4	4	1316.69	902.08
PSM5	2	322.54	314.79
PSM6	3	ND, above 1500	N/A
PSM7	4	ND, above 1500	N/A
PSM8	6	82.34	62.71
PSM9	11	437.89	113.80
PSM10	5	7.26	1.95
PSM11	9	15.93	6.57
PSM12	16	14.16	3.91
PSM13	8	62.21	27.78
PSM14	5	ND, above 1500	N/A
PSM15	3	ND, above 1500	N/A

^a^ *N*, the number of soils averaged; ND, not detectable, the machine could not read beyond 1500 colonies; PSM, phosphorus‐solubilizing microorganism; SEM, standard error of means. Abundance in soil was only determined for PSMs isolated through the “soil‐direct” method.

### 4.3 PSM Isolate Diversity by Locality and Land Use

**Figure 1:**
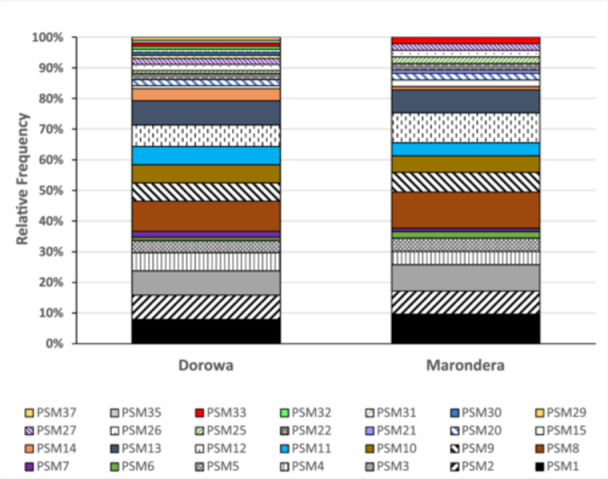
PSM diversity (relative frequency by number of PSMs in each area) in Dorowa and Marondera. PSM, phosphorus‐solubilizing microorganism.

**Figure 2:**
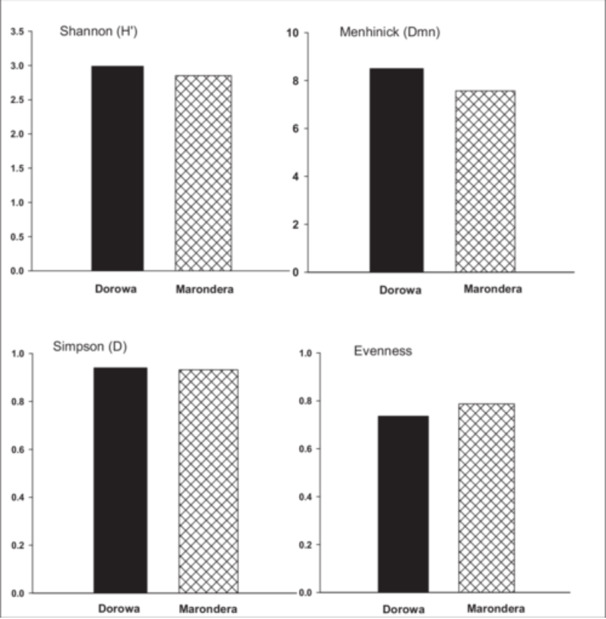
PSM diversity based on the Shannon and Menhinick indices in Dorowa and Marondera. PSM, phosphorus‐solubilizing microorganism.

A total of 37 PSM isolates were obtained, consisting of three fungal isolates and 34 (92.5%) bacterial isolates. Of the total 37 microbial isolates, PSM1–PSM15 were isolated directly from soil, isolates PSM16–PSM37 were obtained from rhizosphere soil of cowpea as a bait plant without any additional nutrients on the different soils. Overall, the number of PSMs isolated from soils sampled along the RP mine belt (Buhera district) was higher than those isolated from selected farmers' fields in Marondera district, as shown in Figure 1 and confirmed by the Taxa‐S values (Dorowa‐27 and Marondera‐22). PSM diversity was higher in Buhera than Marondera as indicated by the Shannon, Simpson, and Menhinick indices. However, species evenness was higher in Marondera district than Buhera (Figure 2). Most (73%) of the PSMs occurred in both districts except PSM28, which only occurred in Marondera and PSM7, 24, 29,30, 32, and 33 occurring only Buhera. PSM8 was the dominant isolate in both areas, followed by PSM1 = PSM2 = PSM3 = PSM13 and > PSM12 (Buhera) and PSM1 = PSM12 > PSM3 > PSM2 = PSM13 (Marondera) (Figure 1).

The largest number of bacterial isolates (20) was obtained from fields previously planted to groundnut (*n* = 2) recording an *H′* index of 3.261. The second‐highest diversity of PSM isolates was recorded from fields previously under maize (*n* = 6) with an *H′* index of 3.259. The lowest PSM isolate diversity (3.185) was recorded in fallow fields or previously cropped to either sunflower or sorghum (Table 4). The diversity trend was also confirmed by the Menhinick index with the lowest index recorded under groundnut (5.096) and the highest (6.11) under cowpea fields, although the two indices had an inverse relationship (Table 4).

**Table 4:** Microbial (phosphorus‐solubilizing microorganism) diversity in relation to cropping history, based on the Simpson, Shannon, Evenness, and Menhinick indices.

Crop/land use	*N*	Simpson		Shannon		Evenness		Menhinick	
		Mean	SE	Mean	SE	Mean	SE	Mean	SE
Cotton	8	0.953	0.0032	3.19	0.0482	0.863	0.0288	5.97	0.163
Cowpea	7	0.952	0.0035	3.19	0.0401	0.866	0.0228	6.11	0.111
Fallow	8	0.953	0.0032	3.19	0.0382	0.863	0.0172	5.97	0.109
Groundnut	20	**0.960**	0.0051	**3.26**	0.0796	**0.931**	0.0102	**5.10**	0.279
Maize	10	**0.959**	0.0033	**3.26**	0.0480	**0.930**	0.0194	**5.60**	0.153
Sorghum	8	0.953	0.0042	3.19	0.0543	0.863	0.0288	5.97	0.163
Sugar bean	13	0.956	0.0056	3.21	0.0702	0.887	0.0165	**5.57**	0.203
Sunflower	8	0.953	0.0032	3.19	0.0482	0.863	0.0239	5.97	0.163
Virgin	16	0.958	0.0059	**3.24**	0.0806	**0.908**	0.0124	**5.46**	0.248
Vlei	11	0.954	0.0033	3.19	0.0566	0.865	0.0157	5.60	0.153

*Note:* Bolded values indicate higher diversity compared with the rest.

### 4.4 Phenotypic Diversity of PSMs: Morphology

The isolates had diverse cultural properties (colony color, elevation, and growth rate), when plated on the selective solid PVK media. Twenty‐eight of the 37 bacterial isolates grew on DRP–PVK agar, with six (PSM1, PSM10, PSM26, PSM29, PSM31, and PSM32) producing distinct concentric rings. Seventeen of the 28 isolates were clear/cream/white in color with some variations, which were identified as glittery, milky white, frosty, or crystal clear. Colony elevation was predominantly raised, while five isolates were dome‐shaped and one isolate, flat with an elevated center. The majority (21 of 37) of the isolates took 5 days to grow on PVK agar. When the isolates were inoculated onto BTB–DRP–PVK agar 13 isolates exhibited fast (2 days) growth, while only 3 (PSM20, PSM21, and PSM35) were slow (7 days) growers, as indicated by a color change from blue to yellow. The investigation was consistent with reported characteristics of fast growth and slow growth of the standard isolates, RHIZO276 (*R. leguminosarum*), and RHIZO267 (*B. diazoefficiens*), respectively. Supporting Information S1 shows the detailed morphological characteristics for the 37 PSM isolates.

### 4.5 Functional Diversity of Selected PSMs: P Solubilization Activity in DRP–PVK Media

All isolates except PSM8 and PSM29 produced a halo zone with diameters ranging between 1.75 (PSM20) and 34.3 mm (PSM14) (Table 5). A large halo zone was recorded for PSM25 and PSM14, followed by PSM13 > PSM22 > PSM27 and PSM31 (*p* < 0.05). There were significant differences in colony diameter, with the top isolates being PSM15 (10.5 mm) > PSM8 > PSM29 > PSM14 > PSM20 > PSM21 > PSM25 > PSM26 > PSM33 (4.5 mm) (Table 5). The PSI ranged between 1 (PSM8 and PSM29) and 15.9 (PSM27), with PSM22, PSM13, and PSM27 having a PSI value above 7 (Table 5).

**Table 5:** Solubilization of P in DRP–PVK media indicated by the PSI at 6 days after incubation.

Isolate code	Colony diameter (mm)	Halo zone diameter (mm)	PSI
PSM1	3.25^abcdef^	5.38^ab^	2.67^a^
PSM2	2.00^abc^	4.25^ab^	3.13^a^
PSM3	1.75^abc^	2.75^ab^	2.58^a^
PSM4	4.23^bcdef^	8.73^ab^	4.00^a^
PSM5	4.23^bcdef^	8.73^ab^	4.00^a^
PSM6	2.50^abcd^	2.00^ab^	1.80^a^
PSM7	2.75^abcde^	5.50^ab^	3.03^a^
PSM8	9.45^1^	0.00^a^	1.00^a^
PSM9	1.50^ab^	2.50^ab^	2.67^a^
PSM10	2.50^abcd^	4.50^ab^	2.71^a^
PSM11	1.00^a^	5.50^ab^	6.50^a^
PSM12	4.00^bcdef^	7.75^ab^	3.08^a^
PSM13	3.25^abcdef^	23.0^bcd^	8.11^ab^
PSM14	8.25^hi^	34.3^d^	5.16^a^
PSM15	10.5^i^	4.50^ab^	1.43^a^
PSM20	8.00^ghi^	1.75^a^	1.22^a^
PSM21	5.75^fgh^	7.00^ab^	2.31^a^
PSM22	3.25^abcdef^	21.0^abcd^	9.28^ab^
PSM25	5.50^efgh^	31.5^cd^	7.04^a^
PSM26	5.25^defg^	2.00^ab^	1.38^a^
PSM27	1.00^a^	13.1^abc^	15.9^b^
PSM29	9.00^i^	0.00^a^	1.00^a^
PSM30	4.23^bcdef^	8.73^ab^	4.00^a^
PSM31	4.00^bcdef^	17.3^abcd^	4.88^a^
PSM32	2.00^abc^	6.25^ab^	4.13^a^
PSM33	4.50^cdef^	4.50^ab^	2.00^a^
PSM35	4.23^bcdef^	8.73^ab^	4.00^a^
PSM37	2.50^abcd^	7.00^ab^	4.00^a^

*Note:* Significant differences at *p* < 0.005. Different letters within a column show significantly different values.

Abbreviations: DRP, Dorowa Rock Phosphate; PSI, Phosphate Solubilization Index; PSM, phosphorus‐solubilizing microorganism; PVK, Pikovskaya.

### 4.6 Biochemical Characteristics

Sixty‐four percent of the isolates were Gram‐positive rods, whilst 29% were Gram‐negative rods. Only 7% of the isolates were Gram‐positive cocci (Supporting Information S2). Most of the isolates were positive for sugar utilization, with 25 isolates (96%) being glucose‐positive and producing gas (96%). Nineteen isolates were positive for sucrose utilization, with over 90% of those isolates producing gas. Sixty percent (60%) of the isolates tested positive for maltose utilization, with 90% of these positive for gas production. Of the 11 isolates which tested negative for maltose, 70% produced no gas. For the fructose test, 16 of the 28 isolates tested negative without gas production, while 83% of the remainder tested positive with gas production (Supporting Information S2). A large proportion of the isolates were negative for the Citrate (82%) and Indole acid (89%) tests. A similar trend was observed for the catalase test, with only 13 isolates being positive (Supporting Information S2).

The standard strains RHIZO276 and RHIZO267 were negative for catalase and citrate and positive for oxidase. The RHIZO276 had a positive indole acid test, while that of RHIZO267 was negative. Supporting Information S2 provides additional information on detailed isolate responses to all the biochemical tests.

### 4.7 Cluster Analyses of Morphological, Biochemical, and P Solubilization Properties of PSMs

**Figure 3:**
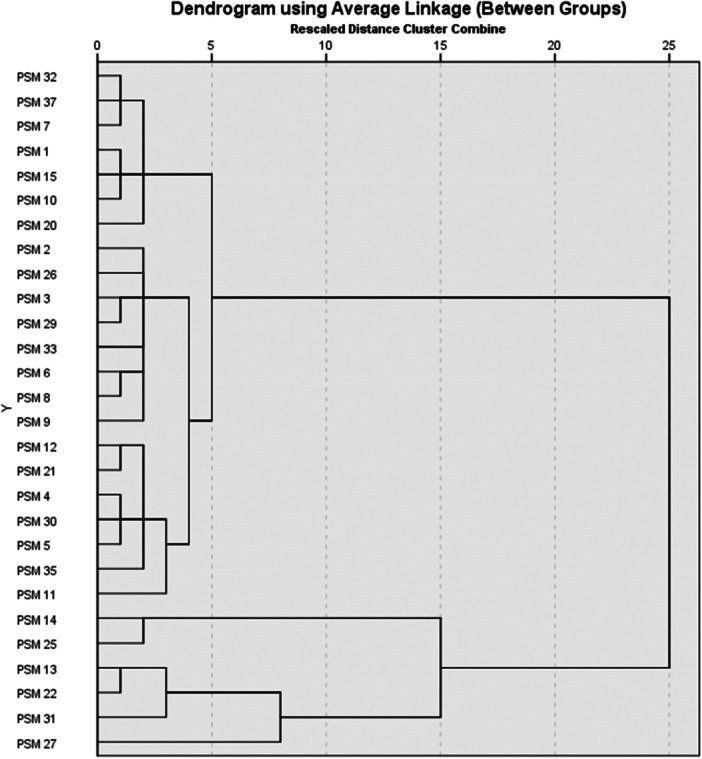
Dendrogram showing the phenotypic relationships generated between PSM isolates from Zimbabwean selected soils. PSM, phosphorus‐solubilizing microorganism.

The diversity of PSMs was not merely a consequence of morphological characteristics, like, color, elevation, and colony size, but was also influenced by the ability to form halo zones, which translated to P solubilization index. Two major clusters were formed with PSM14, PSM25, PSM13, PSM22, PSM31, and PSM27 in group A, characterized by a negative test on lactose, citrate, and indole acid. These six isolates had the highest P solubilization index ranging from 4.88 to 15.9 (PSM27). Group A was further divided into A1 and A2 based on the PSI, with PSM27 demonstrating outstanding performance alone in A2 (Figure 3).

Cluster B comprised the majority of the PSMs with 22 isolates that had highly variable characteristics. This cluster was further divided into B1, B2a, B2b, and B2c. Isolates under B1 included PSM1, PSM7, PSM10, PSM15, PSM20, PSM32, and PSM37 and had white colonies with raised elevation and lower PSI (1.22–4.13), and the rest of the properties were variable. The B2a group comprised PSM2, PSM3, PSM21, PSM26, and PSM33, and these isolates were all glucose positive, citrate‐, indole‐, and Gram‐negative, except PSM2, which was gram positive. Cluster B2b had only three isolates (PSM6, PSM8, and PSM9) with yellow colored, raised colonies, which yielded a positive catalase and maltose test and were medium growers, taking 5 days to reach full growth on DRP–PVK agar.

The PSM6 was indole‐positive, while PSM8 and PSM9 were both indole‐negative. The B2c cluster contained five isolates (PSM4, PSM5, PSM11, PSM30, and PSM35), which were all negative for the citrate, indole, and grams' reaction tests, except PSM11 (Gram‐positive) and PSM30 (indole‐positive).

### 4.8 Phylogenetic Identity and Analyses of PSM Isolates

Nearly full‐length 16S rDNA sequencing revealed that 61% of the PSM isolates belonged to the *Bacillus* genus, with 99%–100% similarity to species listed in Table 6. Within the *Bacillus* genus, the *Bacillus amyloliquefaciens* appeared dominant with PSM7, PSM13, PSM14, PSM22, and PSM25 being closely related to strains within this species. The PSM29, PSM32, and PSM37 were closely related to *Bacillus megaterium*. PSM1 and PSM21 were 99% similar to *Bacillus thuringiensis* strains and PSM27 and PSM 35 similar to other *Bacillus* species not specified. The PSM2 and PSM31 were 100% closely related to strains in *Bacillus cereus* and *Bacillus safensis*, respectively. Three isolates (PSM4, PSM11, and PSM12) were closely related to strains within the *Enterobacter* genus, and two (PSM8 and PSM9) were similar to strains within a *Kocuria* genus. Twenty‐one percent of the isolates were highly diverse in identity with isolates related to *Microbacterium* spp. (PSM5), *Nocardioides* spp. (PSM6), *Paenibacillus* spp. (PSM3), *Acinetobacter seifertii* (PSM10), *Priestia megaterium* (PSM15), and *Klebsiella michiganensis* (PSM26). The 16S rDNA sequencing confirmed the identity of the standard species RHIZO 276 (100% similarity to a *Rhizobia* spp.) and RHIZO 267 (99% similarity to *B. diazoefficiens*).

**Table 6:** Phylogenetic identity details for PSM isolates from Buhera and Marondera, Zimbabwe.

Genetic identity by 16S RNA (99%–100% gene similarity)	Isolate code
*Bacillus thuringiensis* strain FDAARGOS_796 chromosome	PSM1, PSM21
*Bacillus cereus* partial 16S rRNA gene, strain IHBB 5252	PSM2
*Bacillus amyloliquefaciens* strain GXBA‐4 16S rRNA gene	PSM7, PSM14,
*B. amyloliquefaciens* strain 205 chromosome	PSM13, PSM22, PSM25, PSM30
*Bacillus megaterium* strain cqsV16 16S rRNA gene	PSM20
*B. megaterium* strain ROA024 16S rRNA gene	PSM29, PSM32, PSM33, PSM37
*Bacillus* sp. (in Bacteria) strain ATCC 13368 16S rRNA gene	PSM27
*Bacillus* sp. (in Bacteria) strain 49 16S rRNA gene	PSM35
*Bacillus safensis* strain *LSRBMoFPIKRGCFTRI5 16S rRNA gene*	PSM31
*Paenibacillus* sp. AHK180‐5 gene for 16S rRNA	PSM3
*Priestia megaterium* strain FDU301 chromosome	PSM15
*Klebsiella michiganensis* strain Biosolid 27 chromosome	PSM26
Uncultured *Enterobacter* sp. clone F109 16S rRNA gene	PSM4
*Enterobacter* sp. 18A13 DNA complete genome	PSM11
*Enterobacter cloacae* strain AH1 16S rRNA gene	PSM12
*Microbacterium* sp. strain JXJ CY 47 16S rRNA gene	PSM5
*Nocardioides* sp. JS1660 16S rRNA gene	PSM6
*Kocuria* sp. strain ZJFT1123 16S rRNA gene	PSM8, PSM9
*Acinetobacter seifertii* strain WS1 16S rRNA gene	PSM10

Abbreviations: ATCC, American Type Culture Collection; PSM, phosphorus‐solubilizing microorganism; rRNA, ribosomal RNA.

The isolates were deposited into the SPRL‐Microbial Culture Bank at Grasslands Research Station, Marondera, Zimbabwe. The sequences for these isolates were submitted to the NCBI portal on June 16, 2024, and the accession numbers assigned are listed in the Supporting Information material section (S3). The publication is entitled, “Phosphate solubilizing bacteria isolated in Zimbabwe: MAR‐PSB‐ZW,” authored by Kanonge‐Mafaune G, Chiduwa M, and Muchaonyerwa P, under the University of KwaZulu Natal, South Africa.

## 5 Discussion

The infertile and acidic sandy soils used in this study are representative of most cropped soils in Zimbabwe and other parts of sub‐Saharan Africa. They are susceptible to nutrient leaching and need fertility amendments for sustainable productive farming (Mapfumo and Giller 2001; Mungai et al. 2016). The occurrence of PSMs in contrasting niches (agroecological zones and cropping history) in this study suggests that these microorganisms could be found across other similar soils and land uses, although their abundance varied by species, genera, and soil conditions.

It was evident that the soils studied contain PSMs, with the majority being bacteria (Chen et al. 2020), since only three phosphorus‐solubilizing fungal (PSM17, PSM18, and PSM19) species were isolated. The occurrence of fungal PSM18 in both districts in only the fallow, and virgin soils suggested that cropping had a negative effect on this isolate, such that it could not occur in cropped soil. The limited occurrence of fungal PSM17 (only in maize fields) and PSM19 (only in a cowpea field) in Marondera also suggested that these fungal PSMs may only associate with specific agroecosystems, where they may contribute to P or other nutrient cycling. Previous studies have reported that bacteria are more effective than fungi in phosphorus solubilization (Alam et al. 2002; Anand et al. 2016). As a result of this, and the requirements of the facilities under which the work was done, a decision was made to focus on the bacterial isolates, resulting in the characteristics, effectiveness in P solubilization, and classification of the fungal PSMs isolated, to remain unknown.

The higher species diversity in Dorowa versus Marondera, which had higher species evenness, can be explained by the recovery of PSM24, PSM29, PSM30, PSM31, and PSM32, possibly due to differences in rainfall, soil organic matter content, and cultural farming practice differences (Schleuss et al. 2019). The PSMs that occur in only one of the two regions suggested that they could be more sensitive to agroecological conditions (interactions of soil characteristics, climatic conditions, and crop type) than the others (Alori et al. 2017; Pérez et al. 2007). In Dorowa, these PSMs occurred in soils with low organic C (0.31%–0.58%), on groundnut fields with 179 mg P kg^−^^1^ and a maize field which had 41 mg kg^−^^1^ for (PSM30). All these PSMs were recovered by the bait plant method. The PSM28 occurred only in soils from Marondera (NR IIb), only under vlei soil with low P (0.56 mg kg^−^^1^) and high organic C (5.4%). The PSMs occurring only in soils from Dorowa, under NR III, were under specific crops (PSM24, PSM29, and PSM32 only under groundnut and PSM30 only under maize).

The results of this study showed that only soils under maize and groundnut had higher diversity than virgin soil, while those under other crops had lower diversity. This finding suggested that the PSMs could form associations with some plants where they may naturally contribute to P fertility. Bacterial PSMs have been isolated from the rhizospheres of different plants, including chickpea (Cheng et al. 2023), medicinal plants, and soils previously cropped with vegetables, legumes, and cereals dating back from 1903 (Everwand et al. 2017; Fallah 2006). The highest number of PSMs was recovered from a field previously under groundnut and the virgin lands. This can be explained by the benefits of integrating legumes into cropping systems and the rich nutrient base in the undisturbed lands (Kirui et al. 2022). The higher diversity of bacterial PSMs under groundnut (60%) and maize (54%) compared with virgin soils (49%) suggested that these crops promote the diversity of (or are associated with) a diverse group of these bacteria, which they may naturally use to enable P availability. Depending on the effectiveness of the PSMs, the results from our study may suggest that if there is enough P in the soil that the diverse bacterial PSMs under maize and groundnuts could ensure the availability of phosphorus. The low diversity under cotton, sunflower, sorghum, and cowpea suggested that these crops suppress some bacterial PSMs, particularly those that could only be isolated by the bait plant method. Of the PSMs with PSI > 7, sorghum and cotton were associated with only PSM13 (isolated from soil directly), which had among the lowest CFUs in the soils in which it occurred, while cowpea was associated with PSM27 (isolated using cowpea as bait plant). These results suggested that there could be a need to use inoculants when growing these crops in these soils.

The populations of bacterial PSMs isolated in this study (up to 1.5 × 1010 CFU g^−^^1^ soil) were higher than those reported by research teams in Iran (up to 10^7^ cells g^−^^1^ soil) (Silva et al. 2022); in Brazil (2.5–55 × 10^4^ CFU g^−^^1^ of soil) (Kim et al. 1997) and in Kenya (1.30–3.63 × 10^4^) (Kim et al. 1997). Similar to our findings, a higher population density of PSB (8 × 10^5^ and 5.33 × 10^9^) was found in different rhizospheres of vegetable fields (Alia et al. 2013). The population of PSBs varies due to biotic and abiotic factors, as well as other complex biological factors, and host crop preference. Their numbers are not adequately high to compete with other microbial species in the soil or rhizosphere (Jain et al. 2012; Alori et al. 2017; Djuuna et al. 2022). Therefore, bacteria isolated in this study could have been more abundant in relation to the source soils. However, the most dominant or abundant isolates did not produce the biggest halo zone, which corroborates earlier postulations that the most abundant or indigenous soil resident species may not always become the best candidates as biofertilizers (Hussain et al. 2013; Baudoin et al. 2002). Among the isolates with high CFUs, only PSM14, identified as a *B. amyloliquefaciens*, produced a big halo zone and yielded good P solubilization activity in solid DRP–PVK media.

A greater proportion of the isolates were Gram‐positive rods, with differential ability to catalyze the different sugars and varying responses to lactose fermentation in McConkey agar. This agrees with earlier reports (Kim et al. 1997; Satyaprakash et al. 2017), which described P‐solubilizing bacteria as ubiquitous with varying forms and population. The majority of the bacteria isolated could contain enzymes, such as α‐glucosidase (maltase) and β‐fructosidase (invertase). These enzymes hydrolyze maltose and sucrose, respectively (Gachons and Breslin 2016; Patricia and Dhamoon 2022). The fact that the studied PSMs were found to bear variable biochemical characteristics could explain the largely varied P solubilization capacities obtained in DRP–PVK, suggesting that the mechanisms employed by each microbe or microbial cluster were different.

A large proportion of the PSMs recovered from study soils solubilized P in the PVK agar, containing CAP or DRP, producing the characteristic halo zone. When such bacteria are cultured on selective PVK media, they solubilize inorganic P, resulting in the production of acids and the formation of a clear zone (Cheng et al. 2023; Lelapalli et al. 2021). However, some species only developed colonies in the media. Previous studies have shown that further screening in liquid PVK broth or under glasshouse conditions has confirmed their P‐solubilizing ability (Gupta et al. 1994; Behera et al. 2017). In this study, PSMs included both halo zone producers (e.g., PSM13, PSM14, and PSM25) and non‐halo zone producers (PSM8 and PSM29).

The most efficient PSMs were closely related to the *Bacillus* group, specifically dominating was PSM27 (accession number PP919079) closely related to an unknown *Bacillus* spp., with the largest P solubilization index and PSM14 (accession number PP919071) and PSM25 (accession number PP919077) closely related to *B. amyloliquefaciens* species as these two had the largest halo zone diameter. In previous studies, similar *Bacillus* spp., including *B. megaterium* (Rodríguez and Fraga 1999), *B. amyloliquefaciens*, and *B. subtilis* (Zaccardelli et al. 2020) were identified as effective P solubilizers. Therefore, this investigation has revealed potential isolates indigenous to Zimbabwean soils for possible use as PSM inoculants with RP.

Isolates which produced larger halo zones (PSM25_*B. amyloliquefaciens*) or the highest PSI (PSM27_*Bacillus* spp.), were recovered from the cowpea rhizosphere, although two (PSM13 and PSM14_both *B. amyloliquefaciens*) of the isolates recovered directly from soil featured among the top candidates. This agrees with previous findings by several researchers, such as Vazquez et al. (2000) and Kim et al. (1997), who have established that the most metabolically active PSMs are those recovered from the rhizosphere. The wide diversity of the isolated bacteria was confirmed by the BTB–DRP–PVK agar tested, which classified the PSMs into three broad groups as fast, medium, and slow growers, with the majority being fast growers. This validates previous research, which has established that PSMs need between 5 and 7 days of incubation at 28°C ± 2°C for assessment or multiplication (Khan et al. 2009; Zaidi et al. 2009). Among the leading isolates, three (PSM13, PSM14, and PSM25) were classified as fast growers, while one (PSM27) came out as a medium growing isolate. These top isolates did not exceed 5 days to multiply in agar plates and occurred in both Dorowa and Marondera.

The P solubilization index, ranging from 1 to 15.9, confirmed the efficiency of the isolated bacteria in solubilizing P from RP. Although the plate PS index shows the potential of isolates as P solubilizers (Pikovskaya 1948; Richardson Alan and Simpson 2011), existing literature does not provide the critical value for the selection of isolates as the best candidates for PSM bioinoculant development. Eight PSMs were characterized in India and three isolates with a PSI above 4, which ranged from 4.48 ± 0.30 to 4.88 ± 0.69, were selected as the best candidates (Pande et al. 2017). In Nigeria, 39 of the 111 microbes studied were phosphate‐solubilizing microbes (PSMs), whose PSI ranged between 109 and 190. Selected for further study were species identified as *Pseudomonas* spp., *Bacillus* spp., *Micrococcus* spp. (bacteria), *Aspergillus* spp., and *Penicillium* spp. (fungi), with PSI ≥ 140 (Kiprotich et al. 2023). In another study in Kenya, PSMs isolated from fields previously cropped to maize, cowpea, beans, millet, and green gram reached up to 71 with a P solubilization index ranging between 1.14 and 5.88, and the best were selected with PSI > 4 (Kim et al. 1997).

The ability of bacteria isolated in this study to mineralize P in DRP was confirmed and was highly variable. Such bacteria have been discovered and studied, and the P‐solubilizing ability may signify the presence of the enzyme phosphatase (Gachons and Breslin 2016; Kiprotich et al. 2023). The PSMs solubilize P through different mechanisms, including the release of metabolites, such as organic acids, which, through their hydroxyl and carboxyl groups, chelate the cation bound to phosphate, eventually releasing the P (Sagoe et al. 1998) through proton extrusion (Vazquez et al. 2000). The dissolution of P through the production of organic or inorganic acids results in a decrease in pH (Whitelaw et al. 1999; García‐Berumen et al. 2025).

The diversity of the PSMs in terms of their morphological characteristics, biochemical properties, and plate solubilization activity suggested that these organisms varied in phylogenetic identity. This was proven by dendrogram groupings generated through the unweighted pair group method cluster analyses, which did not tally with the phylogenetic groups generated after near‐full‐length 16S rDNA analyses. This could be attributed to within‐genera differences at species and subspecies levels (Patten and Remsen 2017). Previous phylogenetic studies have identified the bulk of soil bacteria, which can solubilize phosphorus as closely related to the following genera *Bacillus*, *Pseudomonas*, *Rhizobium*, *Enterobacter*, *Burkholderia*, *Rhodococcus*, *Arthrobacter*, *Serratia*, *Chryseobacterium*, *Xanthomonas*, *Klebsiella*, *Agrobacterium*, *Azotobacter*, *Erwinia*, *Kushneria*, and *Pantoea* (Sharma et al. 2007; Kirui et al. 2022; Zaidi et al. 2009). The current study confirms the existence of some previously identified groups of bacteria as PSMs with the bulk being related to the *Bacillus* genera, followed by the *Enterobacter* group.

Review of at least 48 studies by Silva et al. (2022) revealed that *Bacillus* (*n* = 22), *Pseudomonas* (*n* = 10), and *Enterobacter* (*n* = 9) were the most dominant genera isolated. These demonstrated the best potential for the development of P‐bioinoculants. No similar studies, on the isolation and evaluation of PSMs had been done in Zimbabwe before the current study. The findings of the current study add to the existing information on the existence of indigenous P‐solubilizing bacteria, dominated by the *Bacillus* and *Enterobacter* genera, together with the *Microbacterium*, *Paenibacillus*, *Kocuria*, *Nocardioides*, *Klebsiella*, and *Acinetobacter* genera, in the studied Zimbabwean soils. Considering the diverse morphological and biochemical characteristics observed in the population of PSMs recovered in the studied soils, and the potential to solubilize DRP demonstrated in plates, it is essential to pursue further research on these isolates to evaluate their efficacy on P solubilization in liquid DRP–PVK broth, under greenhouse or field conditions to determine the potential application as P‐biofertilizers in the real ecosystem. This is the first report of PSB and their phylogenetic designations in Zimbabwe.

## 6 Conclusions and Recommendations

This study has shown that both the arable and the undisturbed lands of Zimbabwe host a diverse population of culturable microorganisms that can solubilize DRP and colonize cowpea rhizosphere. The occurrence and diversity of the PSMs are affected by land use or cropping history, with greater diversity under groundnut and maize than under other crops. The majority of the PSMs occurred in both regions, except PSM17, PSM19 (both fungal), and PSM28 (bacterial) from Marondera only, and PSM24, PSM29, PSM30, PSM32, and PSM33 from Buhera only. These PSMs had different morphological and biochemical characteristics and varied in DRP solubilization activity in plates. The PSMs that belonged to *B. amyloliquefaciens*, and other species in the *Bacillus* genus, differentially exhibited high P‐solubilizing ability, showing the potential for application in future endeavors toward the production of bioinoculants. Considerable work is yet to be done for the selection of the best PSM candidates and testing their efficacy when applied with insoluble RP on various crops under greenhouse and field conditions, with a view to developing P‐solubilizing inoculants and biofertilizers. Considering the relationship between land use and PSM diversity, it may be beneficial to evaluate isolates recovered from a particular cropping history on the specific crop, to generate optimum benefits from the bacteria–plant relationship. More so, it could be valuable to research the most effective method for deployment of the PSM inoculum, to ensure the best results are generated when the PSMs are used under field conditions. Overall, to derive the best results from the coapplication of PSMs and RP, there is a need for further research comparing the potential impact on crop yield improvement against conventional straight P fertilizers, such as the super phosphates and livestock manure.

## Author Contributions

**Grace Kanonge:** conceptualization, investigation, funding acquisition, writing – original draft, methodology, validation, writing – review and editing, formal analysis, data curation, software, resources, visualization. **Mazvita S. Chiduwa:** methodology, writing – review and editing, software, data curation, formal analysis, validation. **Pardon Muchaonyerwa:** conceptualization, funding acquisition, software, supervision, resources, project administration, writing – review and editing, validation.

## Ethics Statement

The authors have nothing to report.

## Conflicts of Interest

None declared.

## Supporting information


**S1:** Morphological characteristics of PSM isolates.

**S2:** Detailed description of biochemical test responses for all the PSM isolates.

S3: Accession numbers assigned to PSM isolates.

## Data Availability

Some of the data that supports the findings of this study are available in the Supporting Information of this article, and other data sets that support the findings of this study are available from the corresponding author upon reasonable request. Additional data on isolates is available in a repository.
