# Supplementary material for: Diversity of Phosphorus‐Solubilizing Microbes Isolated From Different Cropping Systems of Zimbabwe for Use as Biofertilizers With Rock Phosphate
Source: Microbiologyopen. 2025 Oct 13;14(5):e70065. doi: 10.1002/mbo3.70065 (PMC12518785; doi:10.1002/mbo3.70065)
Supplement: Supplementary file 1 — S1: Morphological characteristics of PSM isolates. [file MBO3-14-e70065-s001.docx]

Diversity of phosphorus solubilizing microbes isolated from different cropping systems of Zimbabwe for use as biofertilizers with rock phosphate

*Kanonge Grace^1,3^, Chiduwa. Mazvita. S^2^, Muchaonyerwa. Pardon^3^

*^1 Soil Productivity Research Laboratory (SPRL), Chemistry and Soil Research Institute (CSRI), DRSS,MLAWFRD, P. Bag 3757, Marondera, ZIMBABWE^*

*^2 International Maize and Wheat Improvement Center (CIMMYT), c/o ICRISAT, Chitedze Research Station, Mchinji Road, P.O. Box 1096, Lilongwe, MALAWI^*

*^3 University of KwaZulu Natal, School of Agricultural, Earth, and Environmental Sciences, (SAEES). P. Bag X01, Scottsville, Pietermaritzburg 3201, SOUTH AFRICA^*

**^1^Corresponding author;* [*219085122@stu.ukzn.ac.za*](mailto:219085122@stu.ukzn.ac.za)*; ORCID: 0009-0000-3983-1635*

**S1.** Morphological characteristics of PSM isolates

| **PSM** | **Colour** | **Elevation** | **Growth rate** | **Other unique properties** |
| --- | --- | --- | --- | --- |
| PSM1 | White | Flat | Fast | circular with concentric rings, |
| PSM2 | Clear crystal | Flat | Fast | elevated centre |
| PSM3 | Cream yellow | Dome shaped | Medium | - |
| PSM4 | Clear | Raised | Fast | Circular and flowery |
| PSM5 | Orange yellow | Raised | Fast | - |
| PSM6 | Dark yellow | Raised | Medium | - |
| PSM7 | Red | Raised | Medium | - |
| PSM8 | Light yellow | Raised | Medium | - |
| PSM9 | Bright yellow | Raised | Medium | - |
| PSM10 | White | Dome shaped | Medium | concentric rings |
| PSM11 | Cream | Raised | Medium | flowery ends (rugose) |
| PSM12 | Clear | Flat cumulative | Fast | mushroom shaped |
| PSM13 | Cream-white | Raised | Fast | - |
| PSM14 | Clear | Dome shaped | Fast | Frosty and flashy |
| PSM15 | White | Raised | Medium | With dotted rings |
| PSM16 | Brown | Raised | Medium | Distinct halo zone |
| PSM17 | Grey-white | Raised | Medium | Fungi with halo zone |
| PSM18 | Blue-white | Raised | Medium | Fungi, blue halo zone |
| PSM19 | Grey | Raised | Medium | Fungi |
| PSM20 | Red | Raised | Slow | Isolated minute colonies |
| PSM21 | Cream | Crown Cumulative | Slow | Crown like structure |
| PSM22 | Cream | Raised | Medium | with a rind at edges |
| PSM23 | Mustard (yellow) | Raised | Medium | Isolated minute colonies |
| PSM24 | Dark yellow | Raised | Fast | Dark yellow, fast grower |
| PSM25 | Peach | Raised | Fast | circular |
| PSM26 | Clear | Raised | Fast | with concentric rings |
| PSM27 | Clear | Raised | Medium | with filamentous edges |
| PSM28 | Crystal clear | Raised | Medium | with a ring at the edge |
| PSM29 | Crystal clear | Dome shaped | Medium | with concentric rings |
| PSM30 | Cream | Raised | Fast | With an irregular halo zone |
| PSM31 | White (glittery) | Raised | Fast | with concentric rings |
| PSM32 | White (dotted) | Raised | Fast | With concentric rings |
| PSM33 | Clear | Raised | Medium | Circular minute isolated colonies |
| PSM34 | Cream | Raised cumulative | Medium | - |
| PSM35 | Cream | Raised | Slow | Smooth edges |
| PSM36 | Cloudy | Dome shaped | Medium | - |
| PSM37 | Milky white | Raised | Medium | Isolated minute colonies |
