# Supplementary material for: Diversity of Phosphorus‐Solubilizing Microbes Isolated From Different Cropping Systems of Zimbabwe for Use as Biofertilizers With Rock Phosphate
Source: Microbiologyopen. 2025 Oct 13;14(5):e70065. doi: 10.1002/mbo3.70065 (PMC12518785; doi:10.1002/mbo3.70065)
Supplement: Supplementary file 2 — S2: Detailed description of biochemical test responses for all the PSM isolates. [file MBO3-14-e70065-s003.docx]

Diversity of phosphorus solubilizing microbes isolated from different cropping systems of Zimbabwe for use as biofertilizers with rock phosphate

*Kanonge Grace^1,3^, Chiduwa Mazvita S^2^, Muchaonyerwa Pardon^3^

*^1Soil Productivity Research Laboratory (SPRL), Chemistry and Soil Research Institute (CSRI), DRSS,MLAWFRD, P. Bag 3757, Marondera, ZIMBABWE^*

*^2 International Maize and Wheat Improvement Center (CIMMYT), c/o ICRISAT, Chitedze Research Station, Mchinji Road, P.O. Box 1096, Lilongwe, MALAWI^*

*^3 University of KwaZulu Natal, School of Agricultural, Earth, and Environmental Sciences, (SAEES). P. Bag X01, Scottsville, Pietermaritzburg 3201, SOUTH AFRICA^*

**^1^Corresponding author;* [*219085122@stu.ukzn.ac.za*](mailto:219085122@stu.ukzn.ac.za); *ORCID: 0009-0000-3983-1635*

**S2.** Detailed description of biochemical test responses for all the PSM isolates.

| **Isolate code** | **Grams** | **Glucose** | | **Sucrose** | **Maltose** | **Fructose** | **Catalase** | **Citrate** | **Indole acid** |
| --- | --- | --- | --- | --- | --- | --- | --- | --- | --- |
| PSM1 | +ve-r | +veG+ | +veG+ | | -veG+ | +veG+ | -ve | -ve | -ve |
| PSM2 | +ve-r | +veG+ | +veG+ | | -veGx | +veG+ | -ve | -ve | -ve |
| PSM3 | +ve-r | +veG+ | -veG+ | | -veGx | -veGx | -ve | -ve | -ve |
| PSM4 | -ve-r | +veG+ | +veG+ | | +veG+ | +veG+ | +ve | -ve | -ve |
| PSM5 | +ve-r | +veG+ | +veG+ | | +veG+ | -veGx | -ve | -ve | -ve |
| PSM6 | +r &c | +veG+ | -veG+ | | +veG+ | +veG+ | +ve | -ve | +ve |
| PSM7 | +ve-r | +veGx | +veG+ | | +veGx | -veGx | +ve | -ve | -ve |
| PSM8 | +ve-c | +veG+ | -veGx | | +veG+ | -veGx | +ve | +ve | -ve |
| PSM9 | +ve-c | -veG- | -veG+ | | +veG+ | -veGx | +ve | -ve | -ve |
| PSM10 | -ve-oc | +veG+ | -veG+ | | +veG+ | +veG+ | +ve | +ve | +ve |
| PSM11 | +ve-r | -veG- | +veGx | | -veGx | -veGx | +ve | -ve | -ve |
| PSM12 | -ve-r | +veG+ | +veG+ | | +veG+ | +veGx | +ve | +ve | -ve |
| PSM13 | +ve-r | -veG- | -veG+ | | +veG+ | -veGx | -ve | -ve | -ve |
| PSM14 | +ve-r | +veG+ | +veG+ | | +veGx | +veG+ | -ve | -ve | -ve |
| PSM15 | -ve-r | +veG+ | +veG+ | | -veGx | +veG+ | -ve | +ve | -ve |
| PSM20 | +ve-r | +veG+ | +veG+ | | -veGx | -veGx | -ve | -ve | -ve |
| PSM21 | +ve-r | +veG+ | +veG+ | | +veG+ | -veGx | +ve | -ve | -ve |
| PSM22 | +ve-r | +veG+ | +veG+ | | -veGx | -veGx | +ve | -ve | -ve |
| PSM25 | +ve-r | +veG+ | +veG+ | | +veG+ | -veGx | +ve | -ve | -ve |
| PSM26 | -ve-r | +veG+ | +veG+ | | -veGx | +veG+ | +ve | -ve | -ve |
| PSM27 | -ve-r | +veG+ | +veG+ | | +veG+ | -veGx | -ve | -ve | -ve |
| PSM29 | +ve-r | +veG+ | +veGx | | +veG+ | -veGx | -ve | -ve | -ve |
| PSM30 | +ve-r | +veGx | -veG+ | | +veG+ | +veGx | +ve | -ve | +ve |
| PSM31 | +ve-r | +veG+ | -veG+ | | +veG+ | +veG+ | -ve | +ve | -ve |
| PSM32 | +ve-r | +veG+ | +veG+ | | -veGx | -veGx | -ve | -ve | -ve |
| PSM33 | +ve-r | +veG+ | +veG+ | | +veG+ | -veGx | -ve | -ve | -ve |
| PSM35 | -ve-r | +veG+ | -veG+ | | -veGx | +veG+ | -ve | -ve | -ve |
| PSM37 | +ve-r | +veG+ | +veG+ | | -veGx | -veGx | -ve | -ve | -ve |
| RHIZO267 | -ve-r | +veG+ | +veG+ | | +veG+ | -veGx | -ve | -ve | +ve |
| RHIZO276 | -ve-r | weak+G+ | +veG+ | | +veG+ | -veGx | -ve | -ve | -ve |

*^G+ = positive for gas production, Gx = negative for gas production, +ve = positive, -ve negative, r = rod, c = cocci, oc = oval clustered, NG = no growth^*
