# Supplementary material for: Diversity of Phosphorus‐Solubilizing Microbes Isolated From Different Cropping Systems of Zimbabwe for Use as Biofertilizers With Rock Phosphate
Source: Microbiologyopen. 2025 Oct 13;14(5):e70065. doi: 10.1002/mbo3.70065 (PMC12518785; doi:10.1002/mbo3.70065)
Supplement: Supplementary file 3 — S3: Accession numbers assigned to PSM isolates. [file MBO3-14-e70065-s002.docx]

Diversity of phosphorus solubilizing microbes isolated from different cropping systems of Zimbabwe for use as biofertilizers with rock phosphate

*Kanonge Grace^1,3^, Chiduwa Mazvita S^2^, Muchaonyerwa Pardon^3^

*^1Soil Productivity Research Laboratory (SPRL), Chemistry and Soil Research Institute (CSRI), DRSS,MLAWFRD, P. Bag 3757, Marondera, ZIMBABWE^*

*^2 International Maize and Wheat Improvement Center (CIMMYT), c/o ICRISAT, Chitedze Research Station, Mchinji Road, P.O. Box 1096, Lilongwe, MALAWI^*

*^3 University of KwaZulu Natal, School of Agricultural, Earth, and Environmental Sciences, (SAEES). P. Bag X01, Scottsville, Pietermaritzburg 3201, SOUTH AFRICA^*

**^1^Corresponding author;* [*219085122@stu.ukzn.ac.za*](mailto:219085122@stu.ukzn.ac.za); *ORCID: 0009-0000-3983-1635*

**S3.** Accession Numbers assigned to the P solubilizing bacteria strains studied

| **Isolate code** | **Accession Number** |
| --- | --- |
| PSM1 | PP919059 |
| PSM2 | PP919060 |
| PSM3 | PP919061 |
| PSM4 | PP919062 |
| PSM5 | PP919063 |
| PSM6 | PP919064 |
| PSM7 | PP919065 |
| PSM8 | PP919066 |
| PSM9 | PP919067 |
| PSM11 | PP919068 |
| PSM12 | PP919069 |
| PSM13 | PP919070 |
| PSM14 | PP919071 |
| RHIZO276 | PP919072 |
| PSM15 | PP919073 |
| PSM20 | PP919074 |
| PSM21 | PP919075 |
| PSM22 | PP919076 |
| PSM25 | PP919077 |
| PSM26 | PP919078 |
| PSM27 | PP919079 |
| PSM29 | PP919080 |
| PSM30 | PP919081 |
| PSM31 | PP919082 |
| PSM32 | PP919083 |
| PSM33 | PP919084 |
| PSM35 | PP919085 |
| PSM37 | PP919086 |
| RHIZO267 | PP919087 |

NB. PSM 10, not deposited, sequence not accepted into the Gene bank.
